# Effects of Hot Water and Plant Growth Regulator Treatments on Bud Germination and Pathogen Elimination in Citrus Scions

**DOI:** 10.3390/plants15111674

**Published:** 2026-05-29

**Authors:** Yingzi Zhang, Lisan Sun, Jiangyong Xiao, Hong Chen, Na Li, Dazhi Li, Suming Dai

**Affiliations:** 1 College of Horticulture, Hunan Agricultural University, Changsha 410128, China; zhangyingzi1005@163.com (Y.Z.); 1400033236@stu.hunau.edu.cn (L.S.);; 2 Yuelushan Laboratory, Pomology Variety Innovation Center, Changsha 410128, China

**Keywords:** citrus, pathogen elimination, plant growth regulator, hot water treatment, melatonin

## Abstract

The global citrus industry is under severe threat from devastating diseases, including Huanglongbing (HLB), caused by *Candidatus* Liberibacter asiaticus (*C*Las), and Citrus tristeza disease, caused by *Citrus tristeza virus* (CTV). Establishing efficient pathogen elimination techniques is crucial for developing virus-free citrus nursery systems and helps facilitate the prevention and control of devastating diseases. This study systematically evaluated the multiple effects of hot water treatment and plant growth regulator application on bud germination, pathogen elimination, antioxidant enzyme activities, and *CsWUS* expression in citrus scion. The results showed that moderate hot-water treatment at 40 °C for 5 min promoted bud germination, while higher temperatures or extended durations were inhibitory. Both 6-benzylaminopurine (6-BA) and melatonin (MT) significantly enhanced germination, with 50 mg/L 6-BA and 100 μM MT being most effective. All treatments significantly enhanced pathogen elimination and increased the activities of antioxidant enzymes SOD, POD, and CAT, with melatonin concentrations of 100 μM and above reducing *C*Las and CTV titers by over 93.64% and 96.07%. Furthermore, hot-water treatment and MT, but not 6-BA, upregulated the expression of the virus-resistance-related gene *CsWUS*. A combined treatment of hot water and MT did not yield synergistic benefits. In conclusion, melatonin treatment, particularly at 100 μM, optimally balances high bud germination with effective pathogen elimination, likely through activating antioxidant defenses and CsWUS-mediated immunity. This study provides a practical and efficient strategy for citrus pathogen-free seedling production.

## 1. Introduction

Citrus is one of the most economically important fruit crops globally, providing essential nutrition and generating significant agricultural revenue [1]. However, citrus production is facing unprecedented challenges from devastating diseases, particularly Huanglongbing (HLB, also known as citrus greening) and *Citrus tristeza virus* (CTV). These diseases cause catastrophic annual economic losses to the citrus industry and pose a serious threat to its sustainable development. Huanglongbing, caused by the phloem-limited bacterium *Candidatus* Liberibacter asiaticus (*C*Las), is referred to as the “cancer of citrus”. This highly destructive disease has spread to nearly 50 countries worldwide, resulting in annual economic losses exceeding 10 billion dollars in the global citrus industry [2]. Similarly, *Citrus tristeza virus* (CTV), a member of the *Closteroviridae* family, has caused the death of more than 100 million citrus trees on sour orange rootstocks [3,4,5]. CTV is mainly transmitted through grafting with infected phloem materials and by insect vectors such as the aphid *Toxoptera citricida*. Currently, no effective cure exists for either HLB or CTV, and management strategies primarily focus on prevention, including the use of pathogen-free nursery stock, control of insect vectors, and removal of infected trees [4,6]. Among these strategies, pathogen elimination and the production of pathogen-free planting materials represent a fundamental and effective measure to combat these devastating citrus diseases.

Various techniques have been developed and applied for the elimination of pathogens from citrus, including shoot-tip culture, shoot-tip micrografting, thermotherapy, chemotherapy, and their combinations [7,8]. Among these, methods such as shoot-tip culture and micrografting take advantage of the characteristic that the apical meristem of plants contains very low or undetectable levels of pathogens and are widely used in the clonal propagation of crops. However, these approaches require aseptic conditions and, when applied independently, are often associated with challenges such as browning and vitrification of cultured shoot tips, contamination of explants, high operational technical difficulty, and low pathogen elimination efficiency. Thermotherapy-based pathogen elimination exploits the differential heat tolerance between normal plant cells and pathogens. Under appropriate temperature and duration of treatment, normal plant cells outgrow viral propagation, allowing the newly developed tender tissues to remain pathogen-free. Hot-air thermotherapy has been extensively employed in the asexual propagation of horticultural crops, although it requires extended treatment periods lasting from several weeks to months [9,10]. In contrast, hot water treatment offers advantages of shorter treatment duration, yet it has been less extensively studied in citrus pathogen elimination. Its potential lies in directly inactivating pathogens through short-term heat shock while potentially activating plant defense responses. However, its efficacy against deeply located pathogens is often inconsistent when used alone, and it may negatively affect scion viability. The combination of thermotherapy with micrografting has shown promising results for eliminating CTV and other citrus viruses, but the technique is complex, skill-dependent, and difficult to scale up [11]. Hence, developing an efficient, simple approach that synergistically enhances scion vigor is of great practical significance.

In addition to physical methods for elimination, plant growth regulators (PGRs) have also shown potential in promoting the clearance of pathogens. The exogenous application of PGRs can modulate the endogenous hormonal balance of plants, enhance disease-resistance-related metabolic pathways, and establish systemic induced resistance in the host. These responses could provide protective effects during the early stages of pathogen infection. For example, the cytokinin 6-benzylaminopurine (6-BA) promotes cell division and vitality in meristematic tissues when co-supplemented with naphthaleneacetic acid (NAA) in the culture medium, thereby facilitating pathogen elimination in the shoot apical meristems of tissue-cultured plantlets of apple, strawberry, and sweet potato. Melatonin (MT), a naturally occurring indoleamine compound, has been recognized as a multifunctional plant growth regulator involved in stress tolerance, antioxidant defense, and pathogen resistance [12,13,14]. Recent studies indicate that melatonin enhances plant resistance to pathogens by interacting with salicylic acid (SA)- and jasmonic acid (JA)-mediated defense signaling pathways and modulating plant immune responses [15]. Furthermore, melatonin enhances plant immunity against a variety of pathogens, including viruses. It acts by regulating reactive oxygen species (ROS) levels, activating defense-related gene expression, and improving cell wall integrity. This integrated response establishes a more robust physical and biochemical barrier against pathogen invasion. [16,17,18]. However, research on the application of plant growth regulators such as melatonin for pathogen elimination in woody plants, particularly citrus, is still in its early stages, and the combined effects with physical heat treatments remain unclear.

The physiological and molecular mechanisms underlying successful pathogen elimination often involve complex stress responses. The antioxidant enzyme system, composed of superoxide dismutase (SOD), peroxidase (POD), and catalase (CAT), plays a crucial role in protecting plants from oxidative stress induced by both biotic and abiotic factors [19,20,21]. At the molecular level, WUSCHEL (WUS) is a core member of the WUSCHEL-related homeobox (WOX) transcription factor family, which broadly regulates various stages of plant growth and development [22]. Recent studies have revealed that WUS participates in plant disease resistance responses against viruses. The mechanism may involve WUS maintaining the stem-cell identity and activity of shoot apical meristem cells and regulating downstream defense-related gene networks [23]. This finding explains why meristematic regions remain virus-free even in systemically infected plants. Although the WUS gene family has been characterized in citrus [22], its potential role and dynamic expression patterns during pathogen elimination treatments remain largely unexplored. Elucidating the expression patterns of *CsWUS* during pathogen elimination treatments induced by hot water or plant growth regulators will provide novel insights into the meristem-mediated resistance mechanisms in woody plants.

In this study, we evaluated the effects of hot water treatments and plant growth regulator applications (6-BA and MT) on citrus scion infected with *C*Las and CTV. Specifically, we examined bud germination rates, pathogen elimination efficiency, antioxidant enzyme activities, and *CsWUS* expression to identify optimal treatment strategies for producing pathogen-free citrus plants. This study aims to develop an optimized and efficient approach for pathogen elimination in citrus scions through physical or plant growth regulator treatments without compromising bud germination, and to underly the physiological and molecular mechanisms. The findings will contribute to the development of a simple, stable, and practical technique for producing virus-free citrus plants and provide a valuable reference for pathogen elimination studies in other woody fruit crops.

## 2. Materials and Methods

### 2.1. Plant Materials

Semi-lignified current-year autumn shoots of ‘Newhall’ navel orange (*Citrus sinensis* (L.) Osbeck) were collected from an orchard (25°24′ N, 112°57′ E) in Yizhang County, Hunan Province, China, which was known to be affected by Huanglongbing (HLB) and *Citrus tristeza virus* (CTV). After collection, the shoots were wrapped in moist towels to maintain humidity and transported to the laboratory within one day. Prior to experimental treatments, we confirmed the presence of *Candidatus* Liberibacter asiaticus (*C*Las) and CTV in the plant materials. This initial assessment was based on disease symptoms (Supplementary Figure S1), where HLB infection is primarily characterized by blotchy mottling of leaves [24] and CTV infection by tree tristeza [4]. Pathogen presence was further verified by quantitative real-time PCR (qRT-PCR), following the method described in Section 2.5.

### 2.2. Experimental Treatments

Citrus scions were collected, followed by the removal of leaves and thorns. The scions were scrubbed gently with a soft brush to remove superficial dust, followed by three thorough rinses with sterilized double-distilled water (ddH_2_O) before being used in subsequent experimental treatments. The experiment included a hot-water treatment group with a room-temperature water control, as well as a plant regulator treatment group with a water control. Hot water treatment was performed in a constant-temperature water bath (HSY-12; Shanghai Yuejin Medical Instruments Co., Ltd., Shanghai, China) with a temperature fluctuation of ± 0.5 °C and temperature uniformity of ±0.5 °C. During treatment, the shoots were completely submerged in the hot water. Based on our preliminary experiments, four treatment combinations were established, including exposure to 40 °C for 5 min, 40 °C for 10 min, 45 °C for 5 min, and 45 °C for 10 min. After heat treatment, the scion cuttings were inserted into a vermiculite medium for cultivation. For plant regulator treatments, the effects of 6-benzylaminopurine (6-BA) and melatonin (MT) were tested. MT (Catalog No. M813985-25g, Batch C6463728, ≥98% purity) and 6-BA (Catalog No. B802626-25g, Batch C14369271, ≥99% purity) were purchased from Shanghai Macklin Biochemical Co., Ltd., Shanghai, China. Solutions of 6-BA and MT were prepared by dissolving the powdered reagents in sterile ddH_2_O to the desired concentrations, with no pH adjustment required. Each regulator was applied at four concentrations, 10, 30, 50, and 100 mg/L for 6-BA (correspond approximately to 44.4, 133.2, 222.0, and 444.0 µM), and 50, 100, 200, and 500 µM for MT. The plant regulator solutions were administered by foliar spraying onto scions that had been inserted and cultivated in the vermiculite medium; spraying was performed once daily for three consecutive days.

### 2.3. Cultivation Conditions

All treated scion shoots were maintained in a controlled-environment growth chamber at 26 ± 1 °C, 80% relative humidity, with a 16 h light/8 h dark photoperiod as previous study [25]. Each treatment consisted of 3 replicates with 15 scions per replicate.

### 2.4. Bud Germination Assessment

Citrus scion shoots were observed daily and bud germination was recorded. A bud was considered germinated when new shoot length reached 0.2 cm. The germination rate was calculated as

(1) $$\mathrm{Germination}\;\mathrm{rate}\left(\%\right)=\frac{\mathrm{Number}\;\mathrm{of}\;\mathrm{germinated}\;\mathrm{bud}\;\mathrm{positions}}{\mathrm{Total}\;\mathrm{bud}\;\mathrm{positions}\;\mathrm{on}\;\mathrm{cuttings}}\;\text{×}\;100\%$$

### 2.5. Pathogen Detection

When newly emerged shoots reached approximately 1 cm in length, shoot samples were collected for nucleic acid extraction. Genomic DNA was extracted using the CTAB method [26]. Total RNA was extracted using TransZol Up Plus RNA Kit (TransGen Biotech, Beijing, China) according to the manufacturer’s instructions, and reverse transcription was performed using the TransScript II All-in-One First-Strand cDNA Synthesis SuperMix for qPCR (TransGen Biotech, Beijing, China) following the provided protocol. The concentration and purity (A260/A280 and A260/A230 ratios) of all DNA and RNA samples were measured using a NanoDrop spectrophotometer (TECAN M NANO, Männedorf, Switzerland). The measured purity ratios met the following accepted standards for high-quality nucleic acids: A260/A280 ratios of 1.8–2.0 and A260/A230 ratios > 2.0 for DNA; A260/A280 ratios of 2.0–2.2 and A260/A230 ratios > 2.0 for RNA. The integrity of nucleic acids was further confirmed by 1% agarose gel electrophoresis (Supplementary Figure S2).

Quantitative real-time PCR (qRT-PCR) was performed using ChamQ Universal SYBR qPCR Master Mix (Vazyme, Nanjing, China) to detect *C*Las and CTV titers in emerging shoots. For *C*Las detection, primers *Las*-I-F (5′-CGATTGGTGTTCTTGTAGCG-3′) and *Las*-I-R (5′-AACAATAGAAGGATCAAGCATCT-3′) were used, with *COX* as the reference gene (*COX*-F: 5′-GTATGCCACGTCGCATTCCAGA-3′; *COX*-R: 5′-GCCAAAACTGCTAAGGGCATTC-3′) [27]. For CTV detection, primers *P23*-F (5′-CGTGGATTGYGGTAGAAA-3′) and *P23*-R (5′-CTGAGAYTGYGTATTGTT-3′) were used, with *ACTB* as the reference gene (*ACTB*-F: 5′-CCAATTCTCTCTTGAACCTGTCCTT-3′; *ACTB*-R: 5′-TGACTGATGAGAACTGCCAGAAG-3′) [28]. Amplification was conducted on a Bio Rad C1000 Touch Thermal Cycler with the following program: pre-incubation at 95 °C for 2 min; 40 cycles of amplification at 95 °C for 10 s, 60 °C for 30 s. The total reaction volume was 10 μL, containing 5 μL of 2× SYBR qPCR Master Mix, 0.2 μL of each primer (10 μM), 1 μL template (DNA for CLas detection, cDNA for CTV detection), and 3.6 μL of nuclease free water. Relative pathogen levels were calculated using the 2^−∆∆Ct^ method.

### 2.6. Antioxidant Enzyme Activity Measurement

Samples for antioxidant enzyme activity assays were collected when the newly emerged shoots reached approximately 1 cm in length. The shoots were immediately snap-frozen in liquid nitrogen and stored at −80 °C until analysis. Prior to assay, the frozen tissue was ground to powder in a mortar chilled with liquid nitrogen. Superoxide dismutase (SOD) activity was determined using the nitroblue tetrazolium (NBT) method [29]. Samples were homogenized in pre-chilled phosphate buffer (0.05 M, pH 7.8) and centrifuged; 1.5 mL of the supernatant was collected as the crude enzyme extract. The reaction mixture (3 mL final volume) consisted of 1.5 mL phosphate buffer, 0.3 mL of 130 mM methionine, 0.3 mL of 750 µM nitroblue tetrazolium chloride (NBT), 0.3 mL of 100 µM EDTA-Na_2_, 0.3 mL of 20 µM riboflavin, and 0.1 mL of appropriately diluted enzyme extract. After illumination for 30 min at 25 °C, the reaction was stopped by placing the tubes in darkness. Absorbance was then measured at 560 nm. Peroxidase (POD) activity was measured using the guaiacol method [30]. 0.2 g of plant tissue was homogenized in 1.8 mL of pre-chilled phosphate buffer (0.1 M, pH 6.0) and centrifuged; the supernatant was collected as the crude enzyme extract and kept on ice. The 3.0 mL reaction mixture contained 2.7 mL of 0.1 M phosphate buffer (pH 6.0), 0.1 mL of 2% (*v*/*v*) guaiacol solution, and 0.1 mL of 0.3% (*v*/*v*) hydrogen peroxide solution. After mixing, the change in absorbance at 470 nm was recorded over 1 min. A reaction blank was prepared by replacing the enzyme extract with buffer. Catalase (CAT) activity was determined using the UV absorption method [31]. 0.2 g of tissue was homogenized in 1.8 mL of ice-cold phosphate buffer (0.1 M, pH 6.0) and centrifuged; the supernatant was collected as the crude enzyme extract and kept on ice. The 3.0 mL reaction mixture consisted of 1.9 mL of 0.1 M phosphate buffer (pH 7.0), 1.0 mL of 15 mM H_2_O_2_ solution, and 0.1 mL of appropriately diluted supernatant. After mixing, the decrease in absorbance at 240 nm was recorded over 1 min.

### 2.7. CsWUS Expression Analysis

Samples for *CsWUS* expression analysis were collected from newly emerged shoots upon reaching approximately 1 cm in length. The shoots were immediately snap-frozen in liquid nitrogen and stored at −80 °C until further processing. Prior to RNA extraction, the frozen tissue was thoroughly pulverized in a mortar pre-chilled with liquid nitrogen. Total RNA extraction, reverse transcription, and subsequent qRT-PCR procedure were performed as described in Section 2.5. The citrus WUS homolog (CsWUS) was identified by sequence alignment of citrus WOX family protein sequences [22] with the Arabidopsis WUS protein sequence (At2g17950), which has been shown to inhibit viral protein synthesis [23]. Primers were designed within conserved regions of *CsWUS*: *CsWUS*-F (5′-TAGCAGTGTTGTTGGCGGAT-3′) and *CsWUS*-R (5′-TTGCCATCCGCAGAGTTCTT-3′). Expression levels were quantified by qRT-PCR with *ACTB* as the reference gene.

### 2.8. Statistical Analysis

Data was organized using Microsoft Excel 2016 and analyzed using IBM SPSS Statistics v.22.0. One-way analysis of variance (ANOVA) was performed followed by Duncan’s multiple range test for mean separation at *p* < 0.05. Figures were generated using GraphPad Prism v8.0.

## 3. Results

### 3.1. Effects of Hot Water and Plant Regulator Treatments on Bud Germination

**Figure 1:**
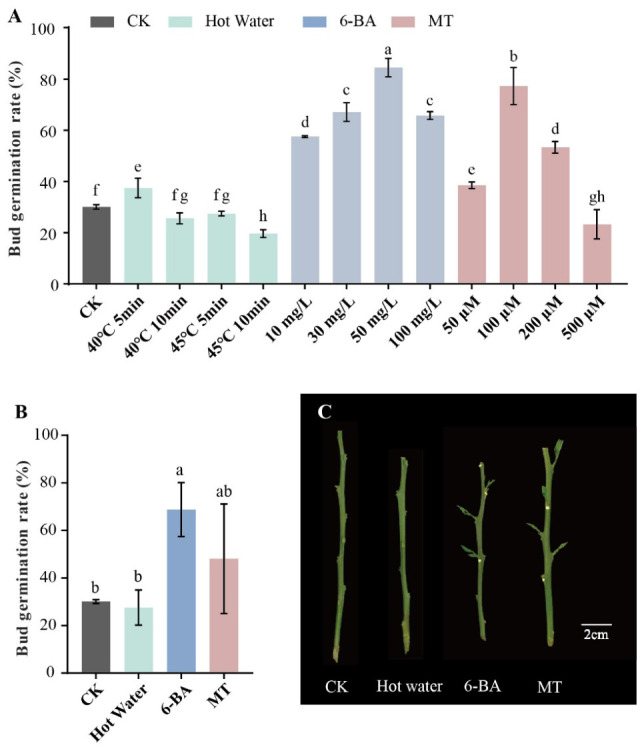
Comparative analysis of bud germination rate under hot water and plant regulator treatments. (**A**) Effects of different hot water, 6-BA and melatonin treatments on bud germination rate of scion. (**B**) Comparison of overall average bud germination rate among different treatment types. (**C**) Representative scion phenotypes under different treatments; the browned area at the base of the scion is a remnant resulting from the cutting propagation process. Different lowercase letters indicate statistically significant differences as determined by one-way analysis of variance (ANOVA), and Duncan’s multiple range test was used for post hoc multiple comparisons at *p* < 0.05. CK, control; 6-BA, 6-benzylaminopurine; MT, melatonin. Data represent means ± SD of three biological replicates.

As shown in Figure 1, bud germination rates were significantly influenced by the applied treatments of hot water and plant regulators. Compared to the control (CK), the 40 °C for 5 min treatment significantly promoted bud germination, showing an increase of approximately 24.6%. However, higher temperature and prolonged treatment duration markedly suppressed the germination rates. Exposure to 45 °C for 10 min resulted in a significantly lower germination rate, averaging 34.5% lower than that of the CK (Figure 1A).

Exogenous application of plant regulators revealed that both 6-BA and MT significantly enhanced bud germination rates. Among 6-BA treatments, 50 mg/L achieved the highest germination rate of 84.46%, which was approximately 180.6% higher than the CK. For MT treatments, 100 µM exhibited the most pronounced effect, with germination rates reaching 77.24%, averaging 156.6% above the CK level. In addition, the promoting effect of bud germination rate showed a pattern of initial increase followed by a decrease at higher concentrations, which showed that high-dose application of plant growth regulators exerts detrimental effects on bud germination. Comparative analysis of the mean bud germination rates revealed significant differences among the treatment groups (Figure 1B). The 6-BA treatment group exhibited the highest mean germination rate, followed by the MT treatment group. Furthermore, the 6-BA group showed a significantly higher mean germination rate than both the hot water treatment and the control group. These results indicate that exogenous plant growth hormone treatments enhance bud germination, whereas hot water treatment did not significantly improve the germination rate.

### 3.2. Effects of Hot Water and Plant Regulator Treatments on Pathogen Elimination

**Figure 2:**
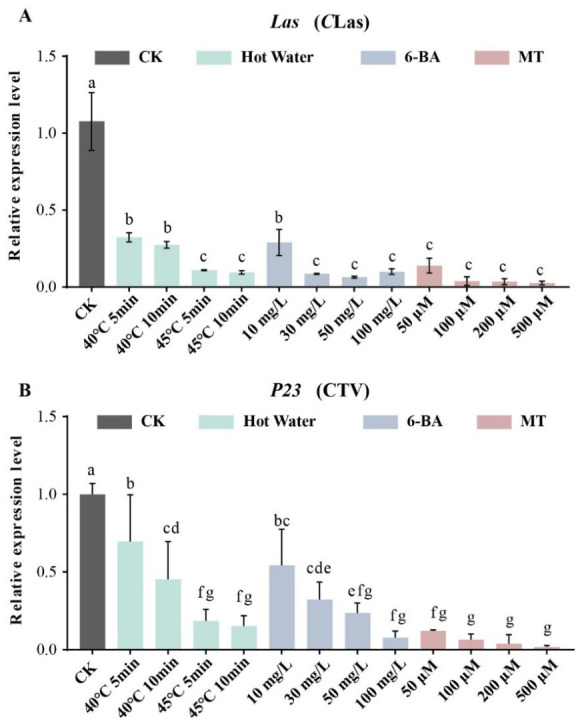
Comparative analysis of the pathogen load of *C*Las and CTV in emerging citrus shoots under hot water and plant regulator treatments. (**A**) Relative expression level of *Las*, which indicates the pathogen load of *C*Las in emerging citrus shoots. (**B**) Relative expression level of *P23*, which indicates the pathogen load of CTV in emerging citrus shoots. Data represent means ± SD of three biological replicates. Different lowercase letters indicate statistically significant differences as determined by one-way ANOVA followed by Duncan’s post hoc test (*p* < 0.05).

To evaluate the effects of hot water treatment and exogenous plant regulator application on pathogen elimination in citrus scion, we comparatively analyzed the level of *C*Las and CTV in emerging citrus shoots by measuring the relative expression levels of the *Las* and *P23*, respectively. The results demonstrated that, compared with the control, both hot water treatment and exogenous plant regulator treatment significantly inhibited the pathogen levels in emerging shoots (Figure 2). Among hot water treatments, the 45 °C treatment most significantly reduced the pathogen loads of both *C*las (91.13%) and CTV (84.89%) than that of CK, with the 10 min treatment at 45 °C showing the highest efficacy. The higher efficacy of the 45 °C treatment likely indicates an optimal balance between the stress intensity required to disrupt pathogen viability and the plant tolerance threshold. Results from exogenous plant regulator treatments showed that both 6-BA and MT significantly reduced the pathogen loads of *C*Las and CTV. Among 6-BA treatments, 50 mg/L was most effective against *C*Las, with a reduction of 93.93%, while 100 mg/L showed the strongest suppression of CTV, achieving a 92.38% decrease. All MT treatments significantly reduced the titers of both pathogens. Specifically, MT at 100 μM and above decreased *C*Las and CTV titers by over 93.64% and 96.07%, respectively, although no significant difference in elimination efficiency was observed among the different concentration groups. The pathogen elimination efficiency plateaued at 100 µM MT, indicating that a further increase in concentration provided no additional benefit.

### 3.3. Effects of Treatments on Antioxidant Enzyme Activity in Emerging Shoots

**Figure 3:**
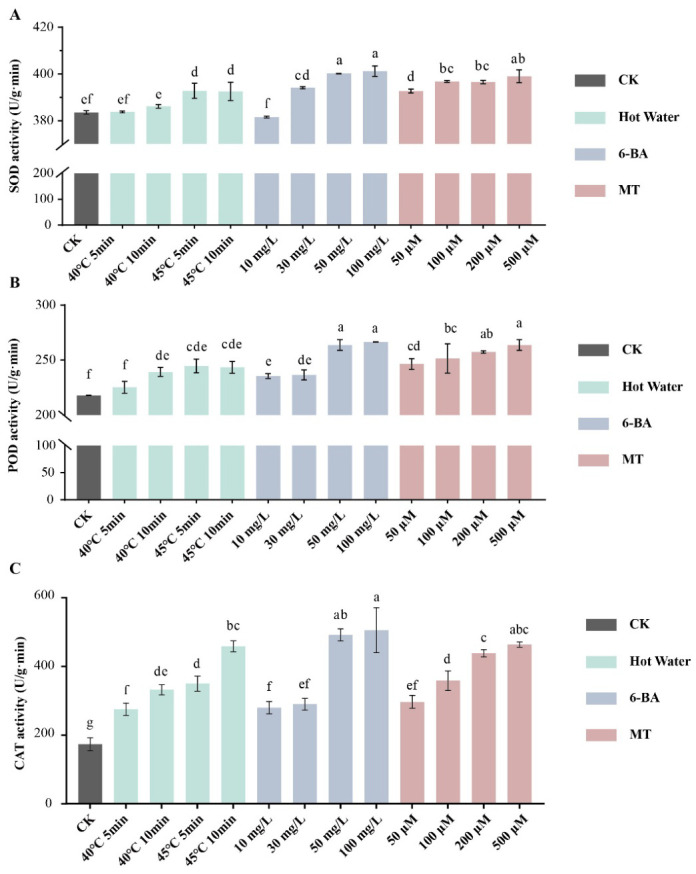
Comparative analysis of antioxidant enzyme activity in emerging citrus shoots under hot water and plant regulator treatments. Comparative analysis of superoxide dismutase (SOD) activity (**A**), peroxidase (POD) activity (**B**), and catalase (CAT) activity (**C**). Data represent means ± SD of three biological replicates. Different lowercase letters indicate statistically significant differences as determined by one-way ANOVA followed by Duncan’s post hoc test (*p* < 0.05).

To investigate the physiological mechanisms underlying pathogen elimination by different treatments, the antioxidant enzyme activities in citrus emerging shoots under various treatment conditions were analyzed and compared (Figure 3). The results showed that hot water treatment at 45 °C significantly increased the activities of both superoxide dismutase (SOD) and peroxidase (POD), with peak activities reaching 392.80 U/g·min and 244.69 U/g·min, respectively. No significant difference in enzyme activities was observed between the 5 min and 10 min treatment durations. Furthermore, hot water treatment significantly enhanced catalase (CAT) activity, with the 45 °C for 10 min treatment showing the greatest promotion, reaching 458.33 U/g·min. The induction of these antioxidant enzymes suggests that heat stress elicits a significant oxidative burst in plant tissues. The upregulation of SOD, POD, and CAT reflect the defense against oxidative damage.

Among exogenous plant regulator treatments, all applications significantly increased the activities of SOD, POD, and CAT, except for 10 mg/L 6-BA, which did not significantly affect SOD activity. The 50 mg/L and 100 mg/L 6-BA treatments exhibited comparable and the strongest effects, with SOD, POD, and CAT activities reaching up to 401.19 U/g·min, 266.56 U/g·min, and 505 U/g·min, respectively. All tested concentrations of MT significantly enhanced antioxidant enzyme activities, and the promoting effect generally strengthened with increasing concentration. The effective enhancement of the antioxidant system by both 6-BA and MT, particularly at higher concentrations, indicates their role in potentiating the plant’s innate defense capacity.

### 3.4. Effects of Treatments on CsWUS Expression in New Shoots

**Figure 4:**
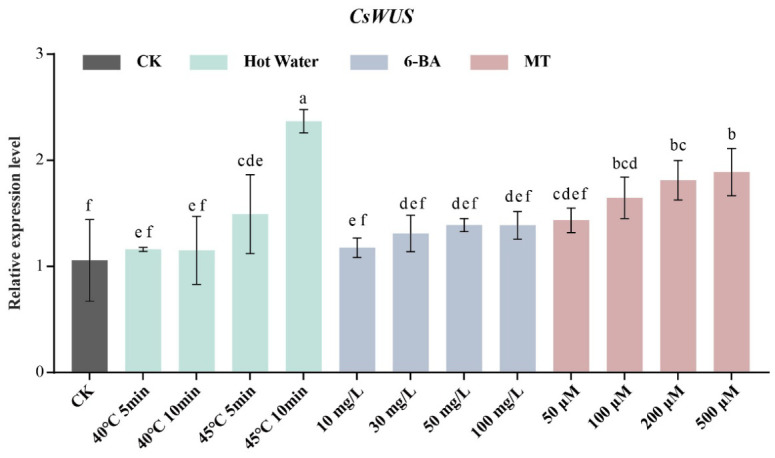
Relative expression levels of *CsWUS* in emerging citrus shoots under different hot water and plant regulator treatments. Different lowercase letters indicate statistically significant differences as determined by one-way ANOVA followed by Duncan’s post hoc test (*p* < 0.05). Data represent means ± SD of three biological replicates. CK, control; 6-BA, 6-benzyl aminopurine; MT, melatonin.

We further compared the expression levels of the virus-resistance-related gene *CsWUS* under different treatment conditions (Figure 4). The results showed that hot water treatments at 45 °C for both 5 min and 10 min significantly upregulated *CsWUS* expression. Compared to the CK, the 10 min treatment of hot water resulted in a 2.24-fold upregulation of *CsWUS* expression. Although 6-BA treatment led to significant pathogen clearance (Figure 2), no detectable effect on *CsWUS* expression level was observed. The dissociation between efficient pathogen elimination and *CsWUS* expression in 6-BA-treated scions implies that this cytokinin may facilitate pathogen clearance through alternative mechanisms, potentially by enhancing general meristematic activity and vitality without directly engaging the CsWUS-mediated transcriptional defense program. Furthermore, the effect of MT treatment on *CsWUS* expression 2varied with concentration. Except for the 50 μM treatment, higher MT concentrations led to a more pronounced upregulation of *CsWUS*. This concentration-dependent induction aligns with the known role of MT as a signaling molecule that modulates defense pathways. The increasing upregulation with higher MT likely reflects a stronger activation of the associated signaling network. These results indicate that the effect of MT treatment on *CsWUS* expression is concentration-dependent, and the differential CsWUS response patterns highlight distinct mechanistic layers underlying pathogen elimination.

### 3.5. Effects of Combined Hot Water and Plant Regulator Treatments

**Figure 5:**
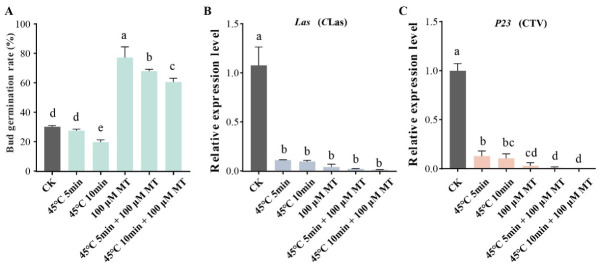
Bud germination and pathogen elimination under hot water and plant regulator treatments. (**A**) Bud germination rate under different treatments. Combined effects on the relative expression level of *Las* (**B**), and the relative expression level of *P23* (**C**) in emerging citrus shoots. Data represent means + SD of three biological replicates. Different lowercase letters indicate statistically significant differences (*p* < 0.05). MT, melatonin.

Given that 100 μM MT treatment showed dual beneficial effects in promoting bud sprouting and eliminating both *C*Las and CTV, we further evaluated the combined effects on pathogen elimination under the optimal conditions of 45 °C hot water treatment and 100 μM MT (Figure 5). Compared to the individual treatments, the combined treatment resulted in an intermediate germination rate. It was significantly higher than that of the individual hot water treatment but lower than that of the individual plant regulator treatment, while still maintaining a value above 60% (Figure 5A). The observed intermediate germination rate likely stems from a balance between the growth-inhibitory effect of sub-lethal heat stress and the growth-promoting, stress-protective role of MT. In addition, compared with the control (CK), the combined hot water and plant regulator treatment significantly downregulated the expression levels of the *Las* and *P23*, indicating enhanced efficiency in eliminating *C*Las and CTV. Furthermore, although the expression levels of *Las* and *P23* in the combined treatment were lower than those in either individual hot water or plant regulator treatment, the differences were not statistically significant. The lack of a statistically significant additive effect suggests that the individual optimized treatments may have each induced a defense response that was near the maximum inducible level under these conditions. These results suggest that the combination of hot water and plant regulator treatments did not produce a pronounced synergistic effect on citrus pathogen elimination.

## 4. Discussion

Thermotherapy has been widely used for eliminating plant pathogens, primarily through high temperatures that inhibit or inactivate pathogenic organisms [8,10]. However, conventional hot air treatment usually requires continuous application for several weeks, which is not only time-consuming but also increases the risk of thermal damage to plant tissues. CTV exhibits relatively high thermal stability in vitro, its thermal inactivation point is approximately 50 °C for 10 min. In our preliminary experiments, treatment at 50 °C severely reduced bud germination. Thus, we applied a short-term hot water treatment under 40–45 °C for 5–10 min. The results showed that hot water treatment significantly reduced the pathogen loads of *C*Las and CTV in citrus, indicating that this method can achieve efficient pathogen elimination while substantially shortening the treatment duration. An effective pathogen elimination technique must strike a balance between elimination efficiency and tissue viability. We propose that the pathogen-elimination effect observed at temperatures below 50 °C is likely indirect. Heat stress may act as a physical stressor that activates plant defense signaling pathways and the antioxidant system, thereby creating a cellular microenvironment unfavorable for viral survival and accumulation. In addition, our experimental results showed that hot-water treatment significantly increased the activities of antioxidant enzymes (SOD, POD, and CAT) in the scions (Figure 3) and markedly upregulated the expression of the virus-resistance-related gene *CsWUS* under the 45 °C treatment (Figure 4). These findings suggest that the elimination of CTV by hot water treatment in this study is closely associated with the activation of the plant’s internal defense system. In the present study, moderate heat treatment exhibited a dual effect on bud germination. Treatment at 40 °C for 5 min significantly promoted the germination rate, whereas higher temperatures or extended durations suppressed germination. This observation aligns with the findings of Divsalar et al. [32], who reported that treating tomato seeds at 56 °C for 30 min caused thermal injury. Ultimately, 45 °C for 5 min was identified as the optimal condition in this study. It maximized the clearance of *C*Las and CTV without significantly inhibiting shoot sprouting, demonstrating a sound balance between pathogen elimination and tissue viability.

Exogenous application of plant regulators is well-established to promote bud outgrowth by stimulating cell division, overcoming apical dominance [33,34], and improving nitrogen assimilation and transport [35]. In our study, treatments with 10–100 mg/L 6-BA and 50–200 µM MT significantly enhanced bud germination rates, which is consistent with the growth-promoting function of these plant regulators. In addition to promoting germination, treatments with 6-BA and MT significantly reduced the pathogen load of *C*Las and CTV in the buds, suggesting that exogenous plant regulators may also enhance systemic disease resistance in plants. This observation aligns with the conclusions of several existing studies. For instance, MT treatment can reduce the occurrence of Rice stripe virus [36], foliar application of MT or salicylic acid decreases the pathogen load of Huanglongbing in sweet oranges [37], and the combined use of 6-BA and IAA facilitates the obtention of pathogen-free seedlings from meristems [38]. Together, these results indicate that beyond regulating growth, plant regulators can also assist in pathogen clearance by inducing resistance responses. In addition, we found that the promotion of bud germination by exogenous plant growth regulators at low concentrations contrasted with its inhibition at high concentrations, which is a common concentration-dependent effect [11]. The suppression of germination following high-dose plant growth regulator treatment may be attributed to the disruption of endogenous hormone homeostasis, excessive induction of reactive oxygen species, and direct cytotoxicity to meristematic cells [11]. For instance, studies have reported that melatonin acts as different roles in the regulation of plant growth and development under low and high concentrations, higher melatonin concentrations can inhibit Arabidopsis seed germination by modulating ABA, GA, and auxin signaling pathways [39]. Given that the individual treatments with 6-BA and MT in this study have already shown significant effects in both promoting germination and eliminating pathogens, their subsequent combination with hot water treatment did not yield further synergistic improvement. We propose that this may result from partial overlaps in their modes of action, as both treatments tend to activate similar plant immune and antioxidant responses, leading to functional redundancy rather than simple additive effects. These results suggest that the plant regulator treatments alone have sufficiently mobilized the plant’s growth and defense potential, and the addition of heat treatment did not provide additional enhancement.

The antioxidant enzyme system constitutes one of the core mechanisms by which plants defend against biotic and abiotic stresses, and its activity is often markedly induced by heat shock or plant regulator application [40,41]. During plant immunity, pathogen infection triggers a burst of reactive oxygen species (ROS) [42,43]. The synergistic scavenging system formed by SOD, POD, and CAT can effectively mitigate oxidative damage [44], thereby protecting cellular structure and function, and enhancing the plant’s overall disease resistance. In this study, both hot water and exogenous plant regulator treatments significantly increased the activities of key antioxidant enzymes, including SOD, POD, and CAT, in citrus emerging shoots. Similarly, Ma et al. [42] reported that exogenous gibberellin upregulates the expression of genes related to hydrogen peroxide scavenging, enhancing antioxidant enzyme activities and ultimately alleviating Huanglongbing symptoms. Therefore, the high pathogen clearance rates achieved by hot water and plant regulator treatments in this study are likely closely associated with the effective activation of the antioxidant defense system.

Beyond these fundamental defense mechanisms, specific signaling pathways may also contribute to pathogen elimination. Recent research have showed that *WUS*, a key regulator of plant stem cells, can be induced by environmental signals or hormones, such as light and auxin [45,46]. It confers antiviral immunity by interfering with ribosomal RNA processing and inhibiting viral protein synthesis [23]. In the present study, significant upregulation of *CsWUS* was detected under both 45 °C hot water and MT treatments, suggesting that their pathogen elimination effects may involve a *CsWUS*-mediated resistance pathway. Interestingly, although 6-BA effectively cleared pathogens, it did not induce significant changes in *CsWUS* expression. This indicates that its mechanism of action may be independent of this pathway, operating instead through alternative mechanisms. This divergence reveals that different treatments may activate distinct molecular pathways to induce plant immunity, the specifics of which warrant further investigation.

While the hot water and plant growth regulator treatments developed in this study show high efficacy for pathogen elimination in ‘Newhall’ navel orange, their application to other fruit genotypes or production systems may require careful adaptation. The optimal treatment parameters, such as the temperature and duration of thermotherapy, the type and concentration of plant growth regulator, are likely to be species- and cultivar-specific [10]. Different perennial plants vary in their heat tolerance and physiological responses to applied regulators. Moreover, the spectrum of pathogens and their intrinsic tolerance to treatments can differ between citrus varieties and other fruit crops, potentially influencing overall eradication efficiency. In grapevine, thermotherapy combined with Ribavirin application is commonly used to eliminate leafroll-associated viruses, while prolonged heat treatment followed by shoot-tip micrografting has been employed for multi-virus clearance in virus-free plant propagation [47,48]. The combination of meristem heat treatment and exogenous hormone application has proven effective in almond trees infected with viruses [49]. These findings illustrate that successful strategies are typically tailored to the specific host–pathogen system and reinforce the view that the methodology developed here for citrus may need to be optimized for different perennial crops. Future studies could systematically screen the tolerance thresholds of different crops to plant growth regulators. The practical efficacy of this approach should also be assessed in key commercial cultivars for pathogen control. Furthermore, combining such an optimized pretreatment with tissue culture techniques like shoot-tip micrografting can provide a technical reference. This integrated strategy will contribute to establishing an effective, sustainable system for producing virus-free nursery stocks and propagating healthy planting materials in perennial crops.

## 5. Conclusions

In summary, this study investigated the effects of hot water and exogenous plant growth regulator (6-BA and MT) treatments on citrus scion bud germination and pathogen elimination. The results demonstrate that both hot water treatment and the application of plant growth regulators effectively eliminated *Candidatus* Liberibacter asiaticus (*C*Las) and *Citrus tristeza virus* (CTV). Exogenous application of 100 μM MT achieved a high bud germination rate while simultaneously clearing both *C*Las and CTV. This dual beneficial effect is likely attributed to the ability of MT to regulate reactive oxygen species (ROS) homeostasis, thereby alleviating stress-induced damage and activating systemic resistance in plants via the upregulation of *CsWUS* expression. These findings provide a simple and effective pre-treatment strategy to produce virus-free citrus nursery plants and offer new insights into the immune regulatory mechanisms of plants under stress.

## Data Availability

The data presented in this study is available on request from the corresponding author.

## Supplementary Materials

The following supporting information can be downloaded at: https://www.mdpi.com/article/10.3390/plants15111674/s1, Figure S1: Phenotypic morphology of autumn shoots and leaves in ‘Newhall‘ navel orange; Figure S2: Agarose gel analysis of RNA quality and integrity (partial samples).
