# Supplementary material for: Effects of Hot Water and Plant Growth Regulator Treatments on Bud Germination and Pathogen Elimination in Citrus Scions
Source: Plants (Basel). 2026 May 29;15(11):1674. doi: 10.3390/plants15111674 (PMC13258924; doi:10.3390/plants15111674)
Supplement: Supplementary file 1 [file plants-15-01674-s001.zip › plants-4320184-supplementary.pdf]

Supplementary Figures

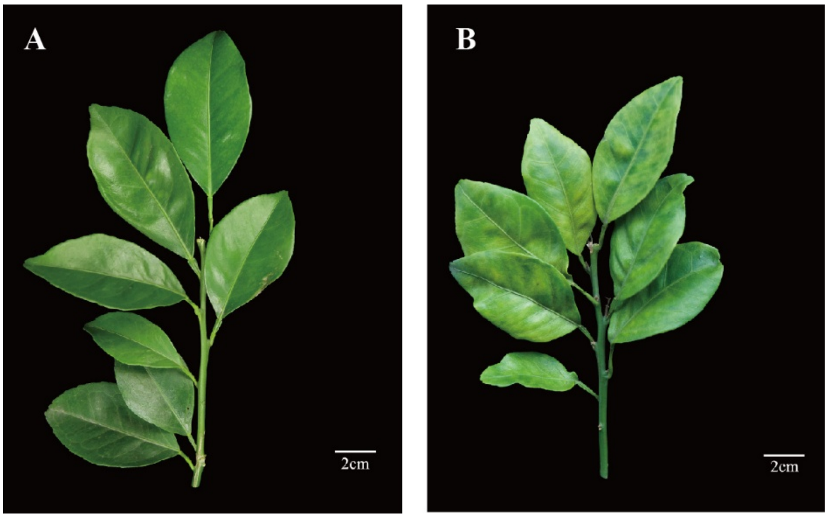

Supplementary Figure S1. Phenotypic morphology of autumn shoots and leaves in 'Newhall' navel orange

(A) Healthy autumn shoots and leaves of 'Newhall' navel orange; (B) autumn shoots and leaves of 'Newhall' navel orange infected with Huanglongbing (HLB).

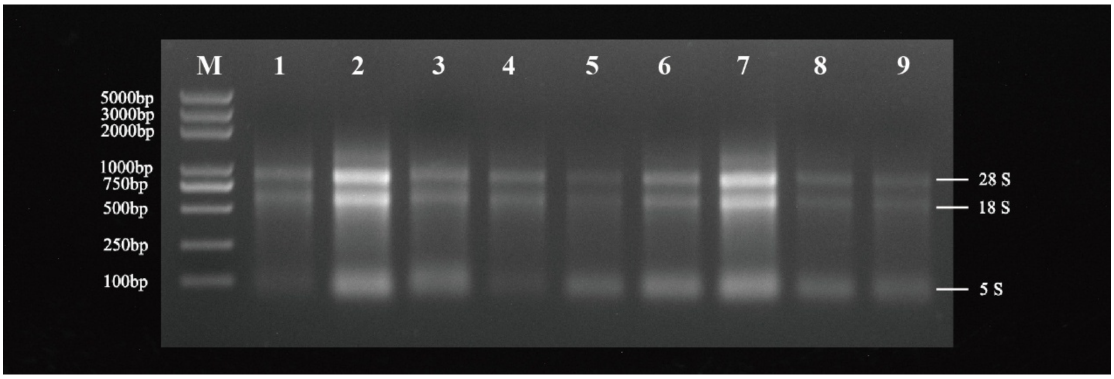

Supplementary Figure S2. Agarose gel analysis of RNA quality and integrity (partial samples)

M, DNA Marker; Lanes 1-9 show representative RNA samples used for subsequent pathogen detection.
